# Functional Foods for Health: The Antioxidant and Anti-Inflammatory Role of Fruits, Vegetables and Culinary Herbs

**DOI:** 10.3390/foods12142742

**Published:** 2023-07-19

**Authors:** Ana S. Fernandes, Cíntia Ferreira-Pêgo, João G. Costa

**Affiliations:** CBIOS—Universidade Lusófona’s Research Center for Biosciences & Health Technologies, Campo Grande 376, 1749-024 Lisboa, Portugal; ana.fernandes@ulusofona.pt (A.S.F.); cintia.pego@ulusofona.pt (C.F.-P.)

The concept of “functional foods” converges topics such as diet, food, health, and disease. Despite a plethora of definitions for functional foods, they are consistently centered on the potential of consuming certain foods and nutrients to better achieve health benefits and decrease the risk of disease.

Fruits and vegetables have been widely investigated due to their health-promoting potential. Likewise, culinary herbs have also been linked to a lower risk of the development of some diseases. These foods present exciting properties, mostly related to the antioxidant and anti-inflammatory actions of their components that may impact the pathogenesis of different diseases such as cardiovascular, neurodegenerative, or cancer conditions. These beneficial effects have been related to their content in various bioactive compounds such as phytochemicals, polyphenols, vitamins, minerals, or organic acids.

This Special Issue featured different scientific articles exploring functional foods, bioactive compounds, and their association with benefits for human health.

Alfheeaid et al. [1] comprehensively investigated three specific species belonging to the Salicornia genus: *S. bigelovii*, *S. brachiata*, and *S. herbacea* and their valuable properties. The nutritional profile of this plant and its impact on human health were described. This plant is an excellent substitute for salt, with direct benefits for human health since it has a lower risk for the development of cardiovascular diseases (e.g., hypertension) and showed protective effects for the liver and kidneys. Salicornia plants present several bioactive compounds, showing antioxidant and anti-inflammatory properties. Moreover, in this review, Alfheeaid et al. discussed the potential application of this plant in the industry and food market, as well as the methods employed for using different Salicornia species as substitutes for salt [1]. 

In another study performed by Lijia Zhang et al. [2], four triterpenoids from *Poriae Cutis*, which is widely used as a dietary supplement and as a food ingredient, were isolated and characterized. The triterpenoid poricoic acid B showed anti-inflammatory activity in RAW 264.7 cells by decreasing the production of different inflammatory mediators, such as TNF-α. Thus, this study showed how a food ingredient can have an important role in health [2].

The health-promoting ability of blackthorn fruit (*Prunus spinosa* L.), traditionally used in nutrition and medicine, was studied by Mirjana Marčetić et al. [3]. A plethora of characteristics, including the total phenolic (TPC), total flavonoid (TFC), and total anthocyanin (TAC) content, as well as the antioxidant, enzyme inhibitory, antimicrobial, and prebiotic activities, were assessed. Twenty-seven phenolic compounds were identified, caffeoylquinic acid being the most abundant compound. The extracts obtained exhibited notable TPCs, TFCs, and TACs, and free radical scavenging and reducing ability. Additionally, enzyme inhibitory effects (e.g., acetylcholinesterase and tyrosinase) were also observed. Blackthorn fruit extracts stimulated the growth of several probiotic microorganisms, namely *Saccharomyces boulardii*. Globally, this study allowed a chemical characterization of blackthorn fruit and demonstrated several beneficial effects of consuming this fruit for human health [3]. 

Wannarat Phonphoem et al. [4] investigated the nutritional composition, the bioactive ingredients, and the antioxidant properties of the pulp and water of Makapuno, a natural coconut cultivar with a unique development of the endosperm, which has a jelly-like appearance. Interestingly, Makapuno pulp showed higher dietary fiber with lower protein and fat content compared to normal coconut pulp. In terms of composition, medium-chain fatty acids were the most abundant component both in pulp and water. The total phenolic, alkaloid, and tannin content was similar in Makapuno pulp compared to those present in mature coconut, while the flavonoid content was lower. Nevertheless, Makapuno water presented higher alkaloid levels compared to normal coconuts. Regarding the promising properties studied, Makapuno (pulp and water) showed antioxidant activities, and the Makapuno water was able to protect against DNA damage. Therefore, this study brought insights into the nutritional and beneficial effects of this fruit, revealing its potential that can be used in the food industry [4].

Erika Ortega-Hernández et al. [5] studied the valuable properties of kale, which is an exceptional source of phenolic compounds, carotenoids, and glucosinolates. In this study, the encapsulation of kale sprouts as a tool to protect and maintain the biological activity of their constituents was performed. The encapsulation efficiency, particle morphology, and storage stability were first assessed. Then, the antioxidant and immunomodulatory properties of the intestinal-digested fraction of the encapsulated kale sprout extracts were evaluated in macrophages and intestinal cells. The encapsulated kale sprouts that grew in the presence of sulfur showed the highest antioxidant and immunomodulatory activities by increasing IL-10 production and inhibiting COX-2 and nitric oxide. Thus, this study demonstrated some beneficial properties of kale for human health and showed that encapsulation can effectively improve phytochemicals’ stability and preserve their bioactivity [5]. 

A study performed by Iswaibah Mustafa and Nyuk Ling Chin [6] addressed the antioxidant properties of ginger (Zingiber officinale Roscoe), a popular culinary herb. Different dried processes and extraction solvents were used, and their efficacy was compared. The drying process showed a positive action on the antioxidant activities of ginger. The ethanol ginger extracts demonstrated higher antioxidant activity and sun-drying was the best method for preserving and increasing ginger quality and the bioactive compounds [6]. This study showed the importance of appropriate drying and extraction methods to maximize the phytochemical content and antioxidant properties from a natural source, in this case, ginger extracts.

A review article authored by Guerreiro et al. [7] offers valuable insights into the potential beneficial effects of polyphenols on various kidney diseases, including acute kidney injury, chronic kidney disease, diabetic nephropathy, renal cancer, and drug-induced nephrotoxicity. The authors also shed light on a crucial factor—the metabolic fate of food bioactive components. This particular aspect has gained significant prominence in recent years due to its implications. The complex pharmacokinetics and extensive metabolization processes that polyphenols and other food bioactives undergo within the human body are vital considerations, particularly when translating in vitro findings into clinical applications.

Since ancient times, the health-promoting properties of foods, particularly vegetables, fruits, and herbs, have been acknowledged. In the present era, this association is no longer purely empirical but rooted in scientific evidence. This Special Issue offers a glimpse into current trends in food research. Mechanistic studies now provide the foundation to substantiate antioxidant and anti-inflammatory claims associated with various food sources. Exciting investigations into novel ingredients and technological processes are underway, aiming to enhance the food industry. This research contributes to unlocking the full potential of food properties, with a specific focus on the remarkable antioxidant and anti-inflammatory role exhibited by fruits, vegetables, and culinary herbs, enabling us to harness the maximum benefits of these natural resources.
